# Targeting invasion-associated proteins *Pf*SUB2 and *Pf*TRAMP in *Plasmodium falciparum*: identification of potential inhibitors via molecular docking

**DOI:** 10.1186/s12879-025-11380-w

**Published:** 2025-08-19

**Authors:** Esther. O. Okafor, Mercy Bella-Omunagbe, Temitope Elugbadebo, Titilope M. Dokunmu, Ezekiel Adebiyi

**Affiliations:** 1 Covenant University Bioinformatics Research (CUBRe), Covenant University, Ota, Nigeria; 2 Department of Biochemistry, Covenant University, Ota, Ogun State Nigeria; 3 Covenant Applied Informatics and Communication Africa Centre of Excellence (CApIC-ACE), Covenant University, Ota, Nigeria; 4 Division of Applied Bioinformatics, German Cancer Research Center (DKFZ), Heidelberg, Germany; 5 African Centre of Excellence in Bioinformatics & Data Intensive Science (ACE), Kampala, P.O. Box 7062, Kampala, Uganda

**Keywords:** Malaria, Subtilisin-like protease 2, Thrombospondin-related apical merozoite protein, *Plasmodium falciparum*, Molecular Docking

## Abstract

*Plasmodium falciparum* subtilisin-like protease 2 (*Pf*SUB2) is responsible for processing *Plasmodium falciparum* thrombospondin-related apical merozoite protein (*Pf*TRAMP). These proteins are essential for asexual blood stage growth and RBC invasion and have, therefore, been identified as potential drug targets. This study predicted the three-dimensional structure of *Pf*SUB2 and *Pf*TRAMP and identified potential inhibitors using molecular docking methods. Five hundred nineteen compounds were docked against both proteins with AutoDock Vina in PyRx. Compounds 139,974,934 and 154,414,021 exhibited better binding affinities when compared to the standard inhibitors, PMSF, which highlights them as suitable inhibitors and potential antimalarials targeting *Pf*TRAMP and *Pf*SUB2. It also highlights 155,204,487 as a compound with dual antimalarial target potential, exhibiting a better binding affinity to *Pf*TRAMP and *Pf*SUB2. The study recommends 139,974,934, 154,414,021, and 155,204,487 as possible compounds for antimalarial drug development.

**Supplementary Information:**

The online version contains supplementary material available at 10.1186/s12879-025-11380-w.

## Introduction

Malaria, caused by the *Plasmodium* genus, is a serious ailment with an estimated 247 million cases reported worldwide in 2021, which marks an increase from the 245 million cases recorded in 2020, which has resulted in 619,000 deaths [1]. Despite notable progress in comprehending the epidemiology of malaria and the existence of multiple therapeutic options, this disease continues to rank among the primary global causes of mortality, with children constituting a significant proportion of those affected by this disease [2]. This increase is attributed to the recent failures in the current antimalarial treatments, including antifolates, artemisinin and artemisinin-based combination therapy (ACT) [3–5]. These drug failures, which are attributed to parasite resistance to antimalarials and the suboptimal effectiveness of licensed drugs, present a significant challenge in the efforts to prevent and control malaria, thus highlighting the pressing need for alternative therapies [6, 7]. The asexual growth of *Plasmodium* begins in hepatocytes, where the parasite infiltrates and proliferates, producing invasive merozoites, which then invade and multiply inside the host’s erythrocytes. The invasion stage of erythrocytes by merozoites is key to the parasite’s growth and survival [8, 9]. This process is driven by the action of at least two proteases: a rhomboid called Rhomboid 4 (ROM4) and the merozoite surface sheddase, a member of the subtilisin-like superfamily called subtilisin-like protease 2 (*Pf*SUB2) [10]. *Pf*SUB2 is essential in the blood stages of the parasite. It carries out protein processing steps to discharge merozoite surface proteins that are important for the egress and invasion of the parasite [11, 12]. *Pf*SUB2 is a sheddase responsible for processing *Plasmodium falciparum* thrombospondin-related apical membrane protein (*Pf*TRAMP) and other invasion proteins such as *Plasmodium falciparum* merozoite surface protein 1 (*Pf*MSP1) and *Plasmodium falciparum* apical membrane antigen 1 (*PfAMA1*). *Pf*TRAMP consists of three distinct domains: a thrombospondin type 1 repeat (TSR) domain, a transmembrane (TM) domain, and a short cytoplasmic tail domain (CTD) that is acidic and lacks the tryptophan residue typically found in thrombospondin related anonymous *protein* (TRAP) family proteins [13]. The cytoplasmic domain of *Pf*TRAMP has been shown to weakly interact with proteins involved in activating the parasite’s actomyosin motor [14]. *Pf*TRAMP is transported as a dimer to the merozoite surface, where it is cleaved by the subtilisin-like protease *Pf*SUB2 at a site between the TSR and the transmembrane domain and is activated, facilitating the attachment and entry of the merozoite to the erythrocytes [14]. *Pf*TRAMP is expressed at the micronemes before relocating to the surface of the merozoite before the initiation of the invasion of the erythrocytes [15, 16]. Studies have shown that *Pf*SUB2 is inhibited by phenylmethylsulfonyl fluoride (PMSF), Dichloroisoprenaline (DCI), and the prodomain structure of *Pf*SUB2, and this also inhibits the shedding of *Pf*TRAMP [12, 15]. *Pf*SUB2 and *Pf*TRAMP, essential for asexual blood stage growth and invasion, have been identified as critical targets for drug development against the parasite [17, 18]. This study aims to identify small molecules using in silico techniques with the capacity to inhibit either *Pf*SUB2 or *Pf*TRAMP or both proteins in *Plasmodium falciparum*. The successful identification of such compounds would make a valuable contribution to the current efforts in malaria control, providing treatment with dual targets, which would reduce the development of antimalarial drug resistance, ultimately reducing the global burden of this disease.

## Methods

### Prediction of the 3D structure of *Pf*TRAMP and *Pf*SUB2

The protein sequence for *Pf*TRAMP was obtained from the UniProt database using the accession number Q8I5M8, which was submitted to I-TASSER (https://zhanggroup.org/I-TASSER/) [19, 20]. The sequence was also submitted to the Robetta (https://robetta.bakerlab.org/) server and modelled using the RoseTTAFold method [21]. The protein sequence for *Pf*SUB2 obtained from UniProt (https://www.uniprot.org/) with the accession number Q8IHZ5 was submitted to the SWISS-MODEL (https://swissmodel.expasy.org/) server and the structure was built using the Alphafold with 99% similarity and Subtilin-like protease from *Dichelobacter nodosus* (3LPD.1.A) with 39% similarity [22].

### Structure evaluation of modelled protein

The quality of the modelled structures was assessed using PROCHECK, ERRAT and VERIFY3D, accessible through the UCLA-DOE LAB– SAVES v6.1 platform (https://saves.mbi.ucla.edu/) and the Molprobity Server (https://molprobity.biochem.duke.edu/) [23–26]. PROCHECK, a protein structure validation tool, analyses stereochemical and geometrical properties such as Ramachandran plot statistics, central chain bond angles and lengths, planarity, Cβ deviation, torsion angles, non-bonded interactions, overall G-factor, and dihedral angles for disulfide bonds [27]. ERRAT analyses the relative frequencies of noncovalent interactions between atoms of various types. It also acts as an extension of the VERIFY3D method, which measures the compatibility of a protein model with its amino acid sequence, which is the 3D-1D compatibility [28]. Thresholds of ≥ 80% are considered acceptable for VERIFY3D and ERRAT scores.

### Ligand library generation

The COACH server, available on the I-TASSER suite, predicts ligands that might bind to a generated protein structure. These ligands were scored with a confidence score (C-score) [19]. The ligand with the highest C-core for *Pf*TRAMP was 2,3-Dihydroxybenzoic acid (DBH) (PubChem ID: 19). Structurally similar compounds to DBH were identified on PubChem using a Tanimoto similarity threshold of 90%, resulting in a ligand library of 328 hits comprising 185 unique conformers which were further screened based on the canonical SMILES resulting in 176 compounds using the Galaxy webserver [29]. Structures similar to PMSF, a known inhibitor of *Pf*SUB2, were also used to build a ligand library, with Tanimoto’s threshold of 90% generating 610 results and 343 unique conformers. In total, the ligand library was made up of 519 compounds.

### Protein and ligand Preparation

To prepare the protein for the subsequent docking calculation, Gasteiger charges were added, and polar hydrogens were added using Chimera Software [30]. The pdb file was converted to pdbqt on PyRx [31]. The ligand sdf files were minimised and then converted to the pdbqt using OpenBabel software [32] on PyRx, which was utilised for the subsequent docking calculations.

### Virtual screening and post-screening analyses

The virtual screening of compounds was carried out using PyRx. The grid box used in this screening for *Pf*TRAMP was set according to the following coordinates: size (x = 25, y = 25, and z = 25), Center (x = 32.8198 Å, y = 1.798 Å, and z = −9.7264 Å) with a grid point spacing of 0.375 Å. The grid box used in this screening for *Pf*SUB2 was set according to the following coordinates: size (x = 25, y = 25, and z = 25), Center (x = −15.8846 Å, y = −27.2194 Å, and z = 15.3262 Å) with a grid point spacing of 0.375 Å. All docking calculations were carried out with an exhaustiveness of 8, According to the resulting binding energies, the top five (5) hits against *Pf*TRAMP, *Pf*SUB2, and the control compounds were selected for the post-screening analysis conducted using Discovery Studio Client 2021.

### Molecular dynamics simulations

Docking-derived protein-ligand complexes were converted into PDB format and uploaded to the CHARMM-GUI Solution Builder to generate all necessary topologies and coordinate files. For each system, the CHARMM36 force field (as implemented in CHARMM-GUI) was used for both protein and ligand parameters. Counterions were added to neutralize the system, and a TIP3P water box extending over the protein and any solute atom was constructed under periodic boundary conditions, following the protocols of Manu et al. and Padiga Seidu et al. [33, 34]. Each solvated complex underwent a two-stage energy minimisation: first, 5,000 steps of steepest descent to relieve bad contacts, followed by 5,000 steps of conjugate gradient until the maximum force on any atom fell below 1,000 kJ· mol¹ ·nm⁻¹. Subsequent equilibration was performed in two phases using GROMACS v.2018.6: (1) NVT equilibration for 12,500 steps (50 ps with a 2 fs time step) at 303 K, maintained by the V‐rescale thermostat; and (2) NPT equilibration for 12,500 steps (50 ps with a 2 fs time step) at 1 bar, controlled by the Parrinello–Rahman barostat. During both equilibration phases, positional restraints (1000 kJ·mol⁻¹·nm⁻²) were applied to all heavy atoms of the protein and ligand to allow the solvent and ions to relax around the complex. Long‐range electrostatics were treated with the Particle Mesh Ewald (PME) method (grid spacing 1.2 Å), and a real‐space cutoff of 1.2 nm was used for both electrostatic and van der Waals interactions, following [35]. All bonds involving hydrogen atoms were constrained using the LINCS algorithm, permitting a 2 fs integration time step. Following equilibration, positional restraints were removed, and each system was subjected to an unrestrained 50 ns production run in the NPT ensemble at 303 K and 1 bar. Trajectories were recorded every 10 ps for subsequent analyses. All MD simulations and trajectory analyses (unless otherwise noted) were performed with GROMACS v.2018.6 package.

### Prediction of ADMET properties of the selected compounds

The absorption, distribution, metabolism, excretion and toxicity (ADMET) properties of the compounds were determined using pkCSM (https://biosig.lab.uq.edu.au/pkcsm/prediction) [36]. The absorption and distribution parameters evaluated were lipophilicity, intestinal absorption, P-glycoprotein substrate, P-glycoprotein inhibitor, BBB permeability and CNS permeability to determine how well these compounds would be absorbed. The metabolism properties evaluated were either if the compounds were substrates of CYP2D6 and CYP3A4 or inhibitors of CYP1A2, CYP2C19, CYP2C9, or CYP3A4 affecting the drug half-life and determining the possibility of drug-drug interactions. Excretion and toxicity parameters include Total Clearance. AMES toxicity, maximum tolerated Dose, hERG I inhibitor, hERG II inhibitor, oral rat acute toxicity and hepatotoxicity.

## Results and discussion

### Evaluation of the 3D structures of *Pf*TRAMP and *Pf*SUB2

**Fig. 1:**
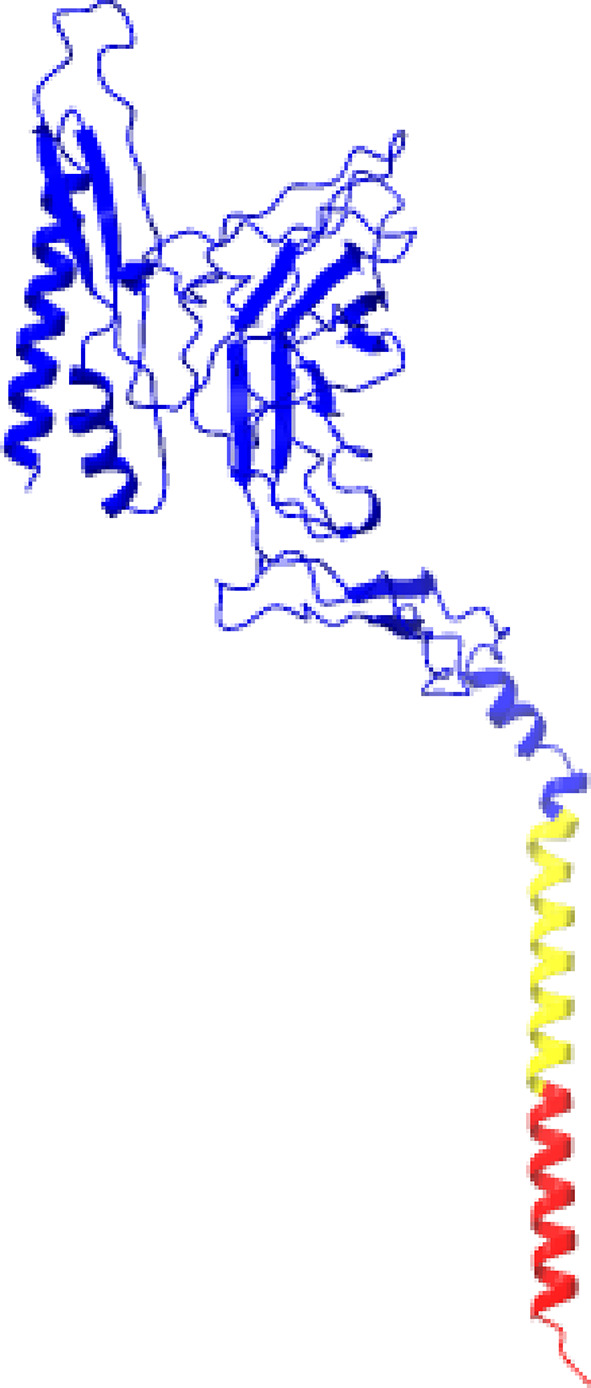
Structure of *Pf*TRAMP; Blue: Non-cytoplasmic domain (1-309); Yellow: Transmembrane Domain (310–331); Red: Cytoplasmic Domain (332–352)

**Fig. 2:**
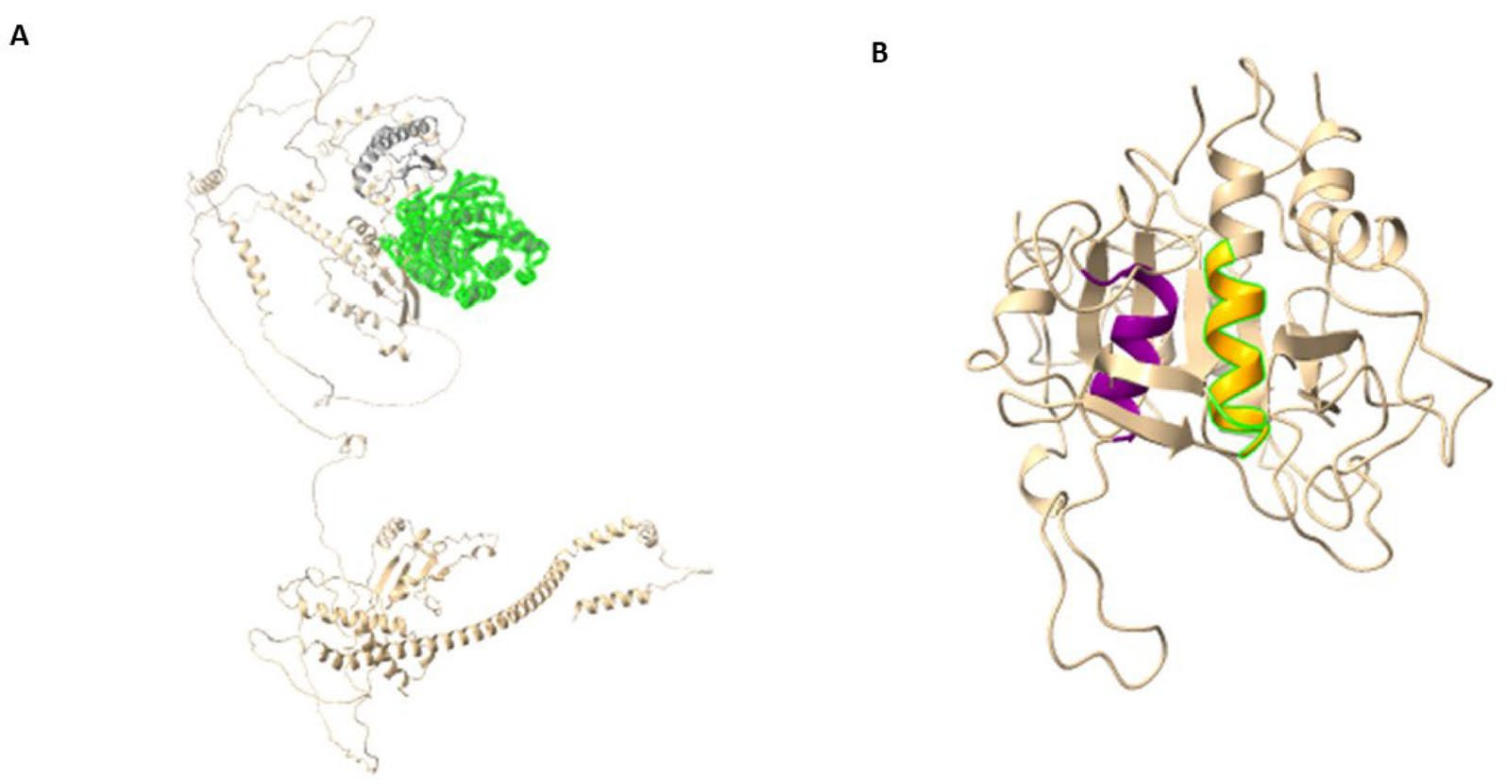
**A**: Structure of *Pf*SUB2; Gray: Pro-domain; Forest Green: Subtilase Domain; **B**: Structure of Subtilase Domain; Purple: Histidine active site; Orange

A total of 11 models were generated for *Pf*TRAMP: 5 from RoseTTAFold, five from I-TASSER, and one from SwissModel. The results from the assessment (Supplementary File 1) show that Model 1 from RoseTTAFold was the best-performing model (Fig. 1). For *Pf*SUB2, the model generated from the 3PLD template represented only the subtilase domain (Fig. 2B) and was used for the docking analysis. In contrast, the AlphaFold template encompassed the full-length protein structure (Fig. 2A). Subsequent analyses were performed using the subtilase domain model derived from the 3PLD template.

### Docking-based virtual screening

The binding affinity of a ligand for a protein increases with an increasing negative value. The results were sorted in order of decreasing negativity. The first five hits with the lowest binding affinity, i.e., the most negative, with *Pf*TRAMP (Table 1) and *Pf*SUB2 (Table 2), were selected for post-docking analysis. This analysis studies the interactions of the ligands with the active site of *Pf*TRAMP and *Pf*SUB2 compared with DBH, PMSF, and DCI.

**Table 1:** The binding affinities of the best hits against *Pf*TRAMP

S/*N*	Compound ID	Structures	Binding Affinity (kcal/mol)
1	139974934		−7
2	139975082		−7
3	135307358		−6.8
4	155204487		−6.7
5	136630885		−6.6
6	DCI		−5.5
7	DBH		−5.2
8	PMSF		−4.9

**Table 2:** The binding affinities of the best hits against *Pf*SUB2

S/*N*	Compound Codes	Structures	Binding Affinities(kcal/mol)
1	154414021		−5.9
2	5919167		−5.9
3	154074555		−5.8
4	88759054		−5.8
5	149253472		−5.7
6	155204487		−5.7
7	DCI		−4.5
8	PMSF		−4.2
9	DBH		−4

### Post-screening analysis of selected compounds against *Pf*TRAMP

**Fig. 3:**
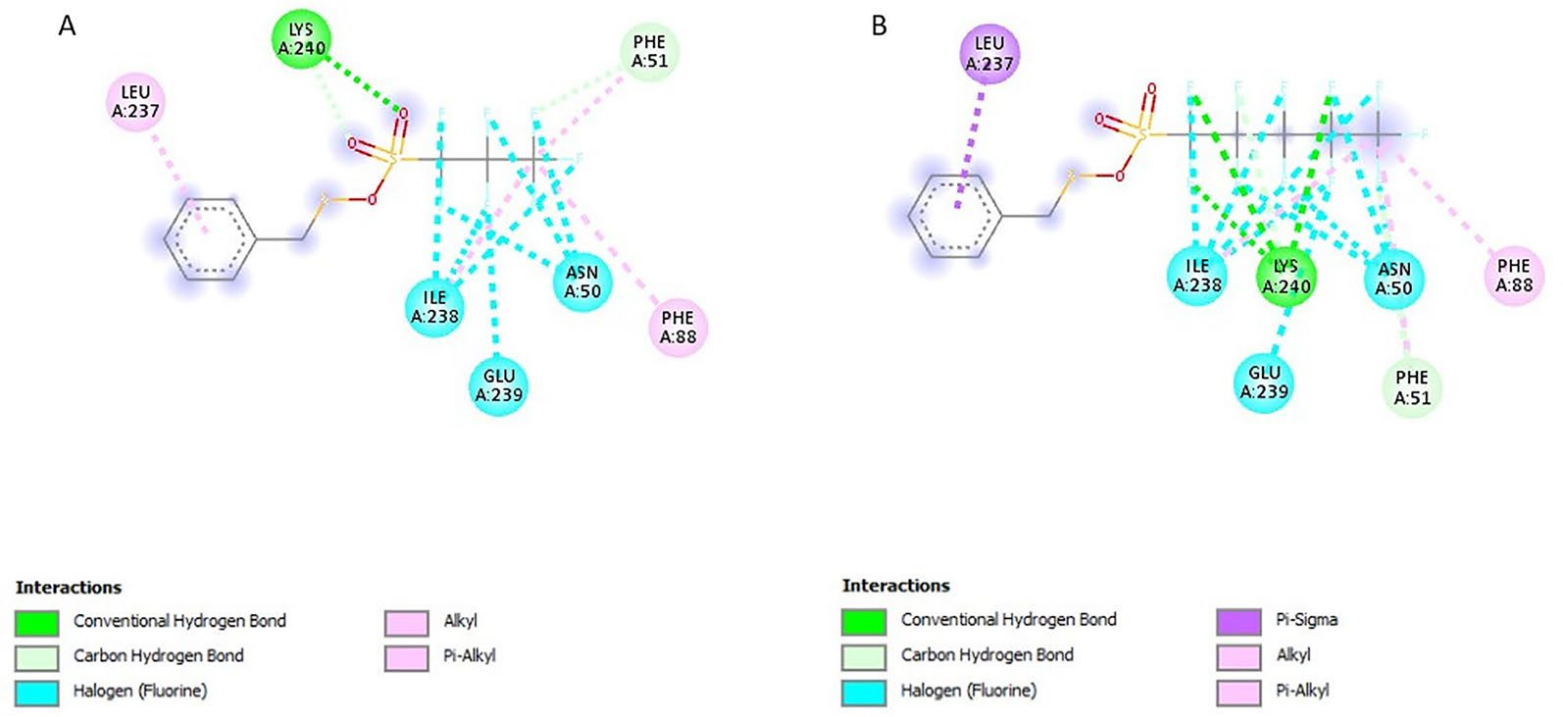
2D structures representing the binding interactions between A: **139974934** and B: **139975082** with *Pf*TRAMP

**Fig. 4:**
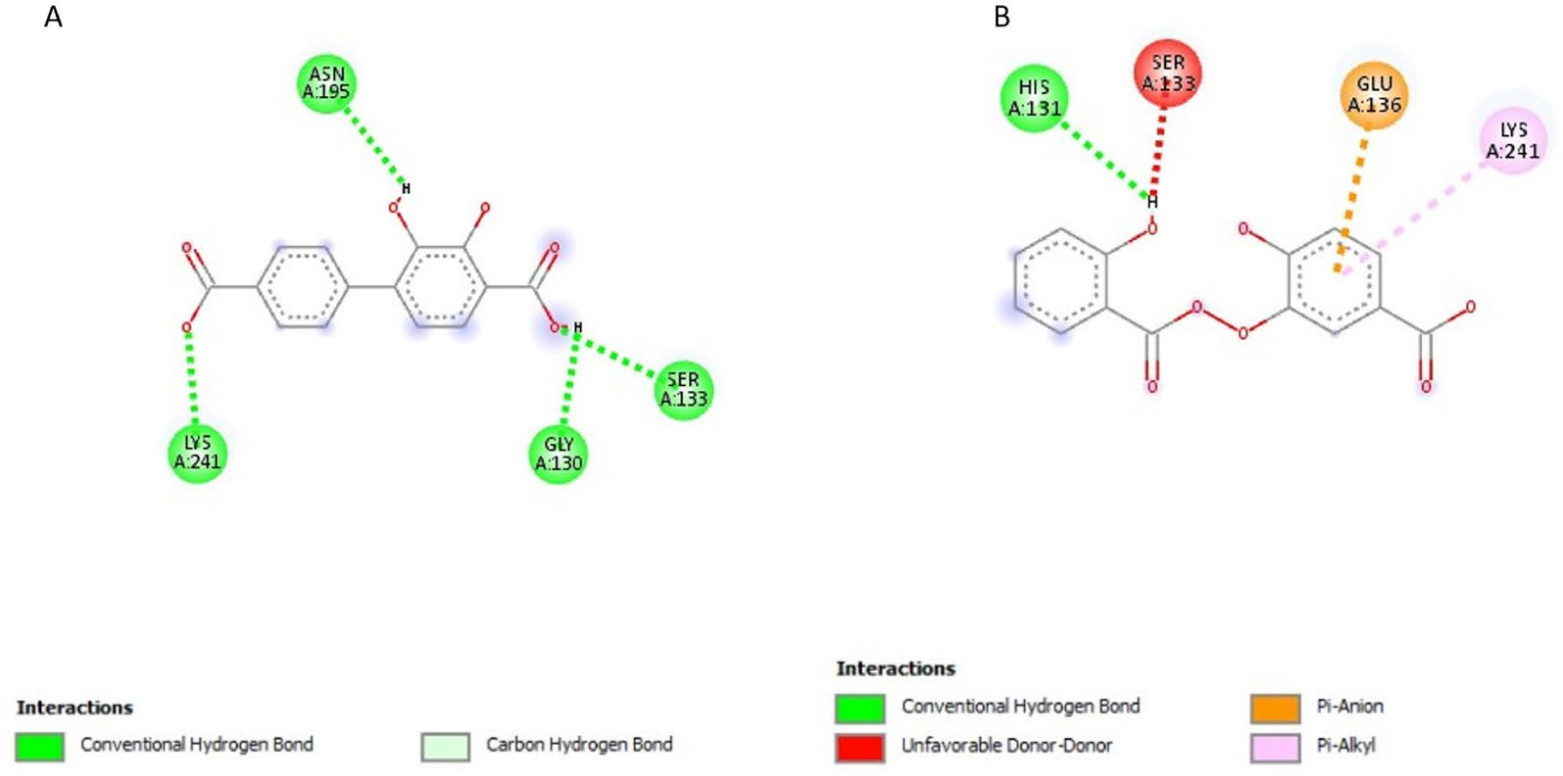
2D structures representing the binding interactions between **A**: **135307358** and **B**: **155204487** with *Pf*TRAMP

**Fig. 5:**
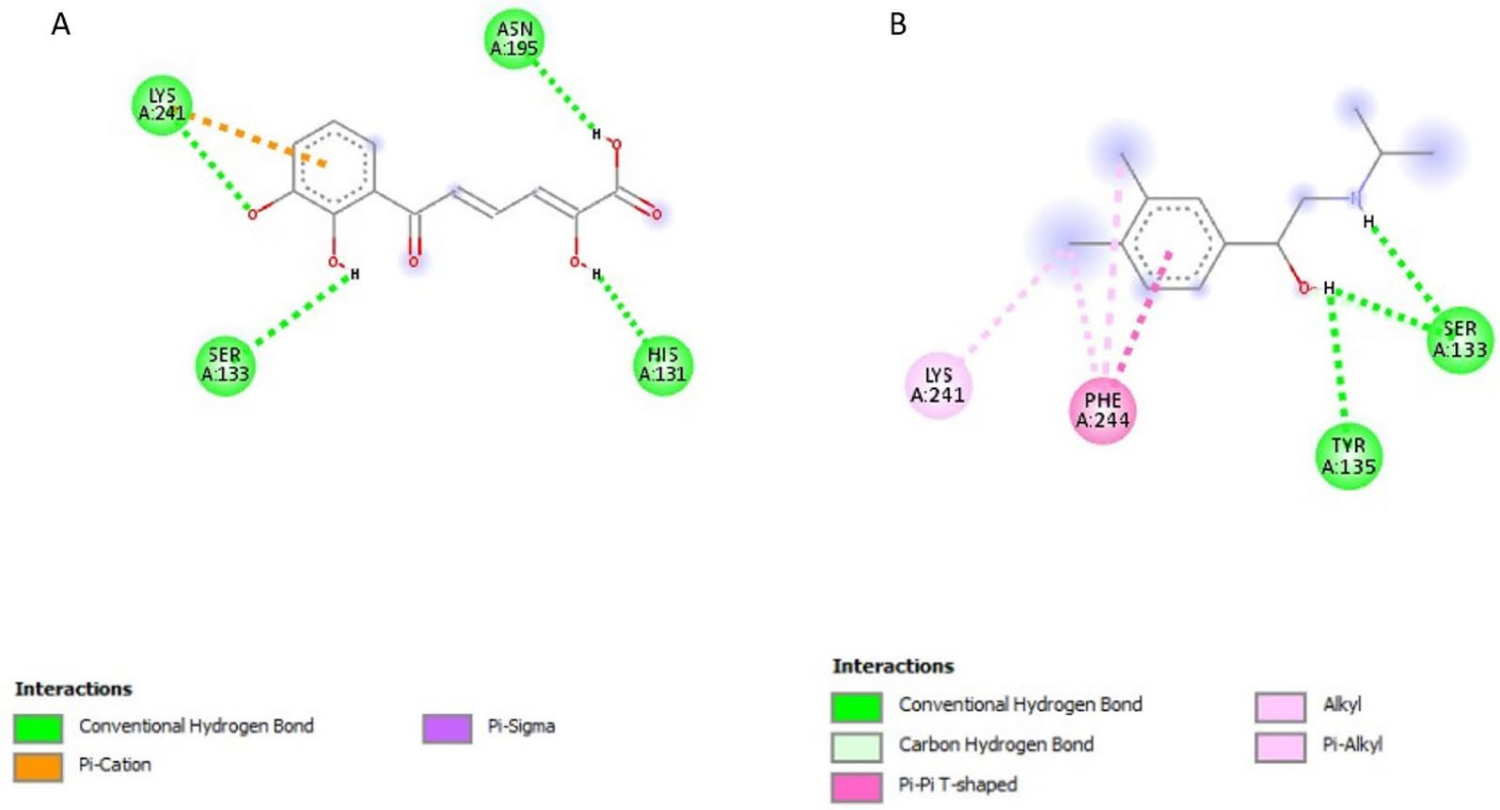
2D structures representing the binding interactions between** A**: **136630885** and **B**: **5806** with *Pf*TRAMP

**Fig. 6:**
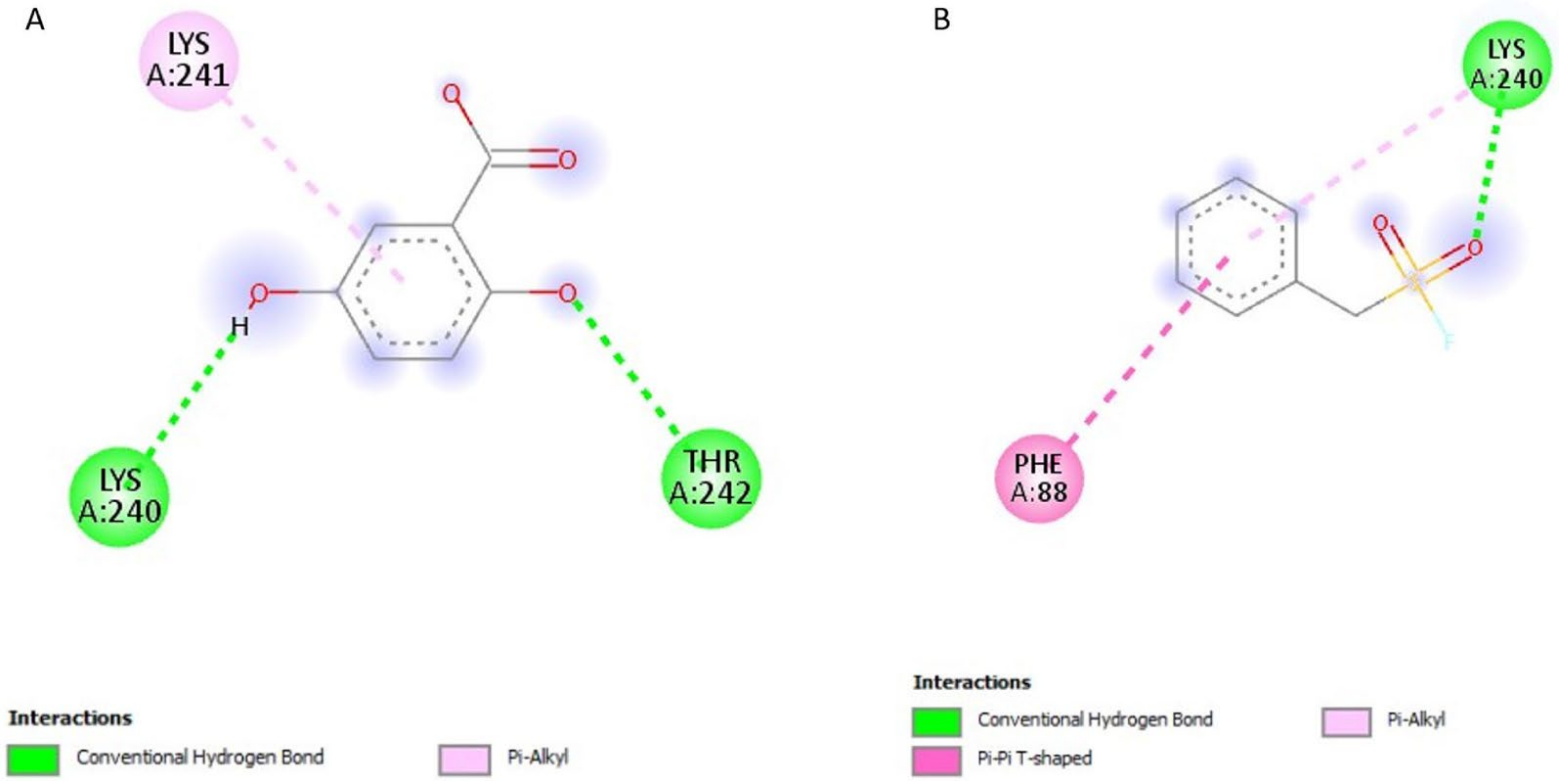
2D structures representing the binding interactions between **A**: **19** and **B**: **4784** with *Pf*TRAMP

The interactions of the ligands in the active site of *Pf*TRAMP were visualised with the Discovery Studio 2021 client software. The interactions are shown in Table 3. The compound 139974934 possessed the lowest binding affinity from this study at −7 kcal/mol and is, therefore, the first hit. It has no Hydrogen Bond Donors (HBD) and has 11 Hydrogen Bond Acceptors (HBA). It formed a conventional hydrogen bond with LYS240, carbon-hydrogen bonds with PHE51 and LYS240, alkyl/pi-alkyl with PHE88, PHE51, ILE238 and LEU237, and halogen interactions with ILE238, GLU239 and ASN50 (Fig. 3A). Compound 139975082 with 0 HBD and 15 HBA formed conventional hydrogen bonds with LYS240, carbon-hydrogen bonds with PHE51 and LYS240, an alkyl/pi-alkyl interaction with PHE88, PHE51, and ILE238, pi-sigma interaction with LEU237 and halogen interactions with ILE238, GLU239 and ASN50 (Fig. 3B). These two compounds, similar in structure to PMSF, showed Compound 135307358 exhibited a binding affinity of −6.8 kcal/mol. It possesses 4HBD and 6HBA and forms conventional hydrogen bonds with ASN195, SER133, GLY130 and LYS241 (Fig. 4A). Compound 155204487 exhibited a binding affinity of −6.7 kcal/mol and formed a conventional hydrogen bond with HIS131, a Pi-Alkyl interaction with LYS241 and a Pi-Anion interaction with GLU136 (Fig. 4B). Compound 136630885 formed conventional hydrogen bonds with LYS241, ASN195, SER133 and HIS131, and a Pi-Cation interaction with LYS241 (Fig. 5A). The reference compounds all exhibited higher binding affinities. Compound 5806 with 2 HBD and 2 HBA exhibited a binding affinity of −5.5 kcal/mol. It formed conventional hydrogen bonds with TYR135 and SER133, alkyl/pi-alkyl interactions with LYS241 and PHE244 and a Pi-Pi T-shaped interaction with PHE244 (Fig. 5B). Compound 19 with 3 HBD and 4 HBA exhibited a binding affinity of −5.2 kcal/mol, formed conventional hydrogen bonds with LYS240 and THR242, and a Pi-Alkyl interaction with LYS241 (Fig. 6A). Compound 4784 also exhibited a binding affinity of −4.9 kcal/mol and possessing 0 HBD and 3 HBA formed conventional hydrogen bonds with LYS240, Pi-alkyl interaction with LYS240 and Pi-Pi T-shaped interaction with PHE88 (Fig. 6B). Compounds 139974934 and 139975082 are similar in structure to PMSF (4784) and exhibited the highest binding affinity when docked against *Pf*TRAMP. These two compounds also showed significant similarity with PMSF in the amino acid residues they interacted with. However, these two compounds had hydrogen bond acceptors > 10, which violates the Lipinski Rule of Five, and these can also be managed through hit-to-lead optimisation techniques. The last three compounds similar in structure to DBH (19) showed similarity in the amino acid residues they interacted with along with DCI (5806). The ligand interactions were all seen to be formed with the non-cytoplasmic domain of *Pf*TRAMP, where the protein’s activity lies, indicating the potential of these compounds to affect the activity of the protein even after cleavage or processing by *Pf*SUB2.

**Table 3:** Ligand interactions of selected compounds with *Pf*TRAMP

S/*N*	Compounds	Hydrogen Bond Donors	Hydrogen Bond Acceptors	Interactions and Bond Lengths
1	benzylsulfanyl 1,1,2,2,3,3,3-heptafluoropropane-1-sulfonate	0	11	Conventional Hydrogen Bond: PHE51 LYS240 Carbon-Hydrogen bonds: PHE51 and LYS240 Pi-Alkyl: PHE88, PHE51, ILE238 and LEU237 Halogen (Florine): ILE238, GLU239 and ASN50
2	benzylsulfanyl 1,1,2,2,3,3,4,4,5,5,5-undecafluoropentane-1-sulfonate	0	15	Conventional Hydrogen Bond: LYS240 Pi-Alkyl: PHE88, PHE51, and ILE238 Pi-Sigma: LEU237 Carbon-Hydrogen Bonds: PHE51 and LYS240
3	4-(4-carboxyphenyl)−2,3-dihydroxybenzoic acid	4	6	Conventional Hydrogen Bond: ASN195, SER133, GLY130 and LYS241
4	4-hydroxy-3-(2-hydroxybenzoyl)peroxybenzoic acid	3	7	Conventional Hydrogen Bond: HIS131 Pi-Alkyl: LYS241 Pi-Anion: GLU136
5	(2Z,4E)−6-(2,3-dihydroxyphenyl)−2-hydroxy-6-oxohexa-2,4-dienoic acid	4	6	Conventional Hydrogen Bond: LYS241, ASN195, SER133 and HIS131 Pi-Cation: LYS241
6	DCI	2	2	Conventional Hydrogen Bond: TYR135 and SER133 Alkyl/Pi-Alkyl: LYS241 and PHE244 Pi-Pi T-shaped: PHE244
7	DBH	3	4	Conventional Hydrogen Bond: LYS240 and THR242 Pi-Alkyl: LYS241
8	PMSF	0	3	Conventional Hydrogen Bond: LYS240 Pi-Pi T-shaped: PHE88 Pi-Alkyl: LYS240

### Post-screening analysis of selected compounds against *Pf*SUB2

**Fig. 7:**
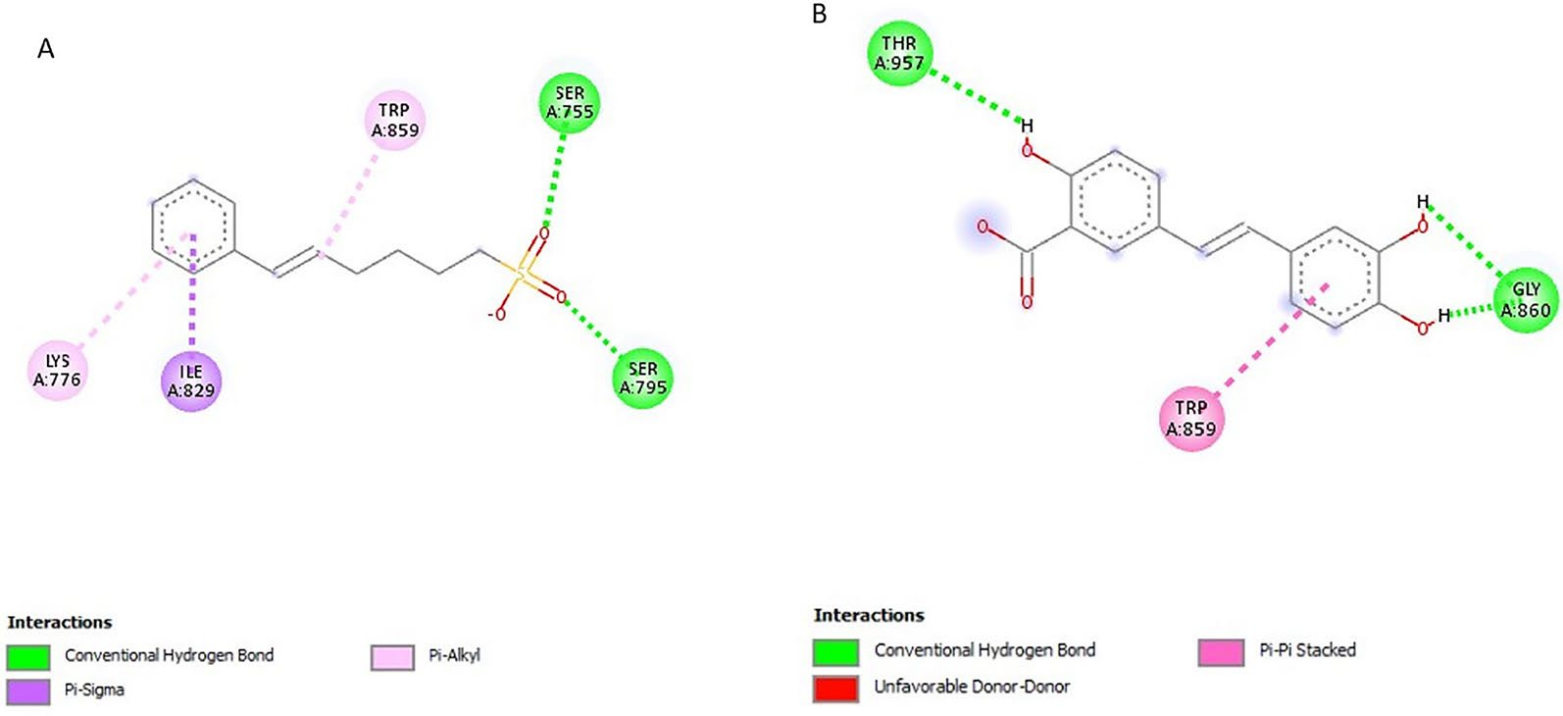
2D structures representing the binding interactions between **A**: **154414021** and **B**: **5919167** with *Pf*SUB2

**Fig. 8:**
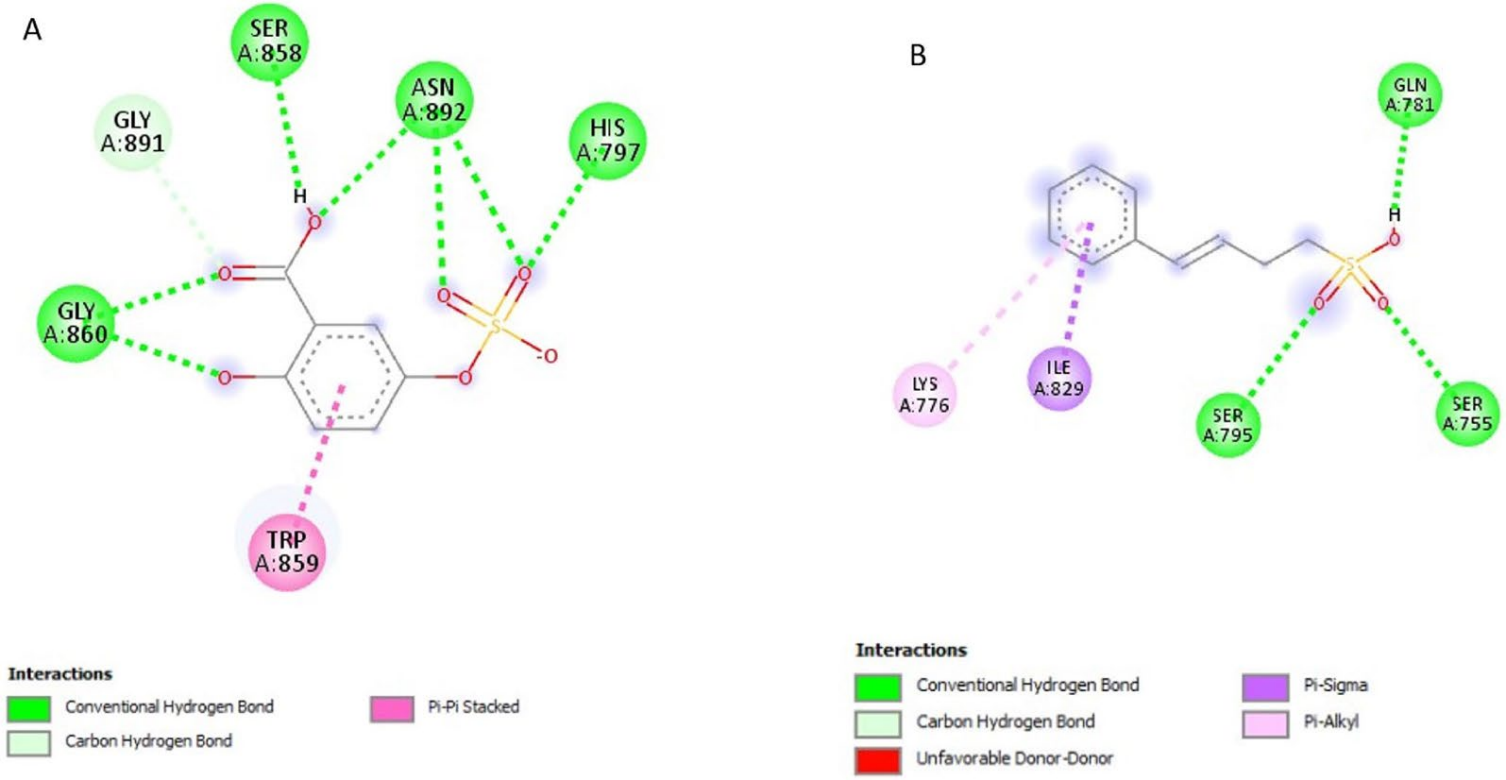
2D structures representing the binding interactions between **A**: **154074555** and **B**: **88759054** with *Pf*SUB2

**Fig. 9:**
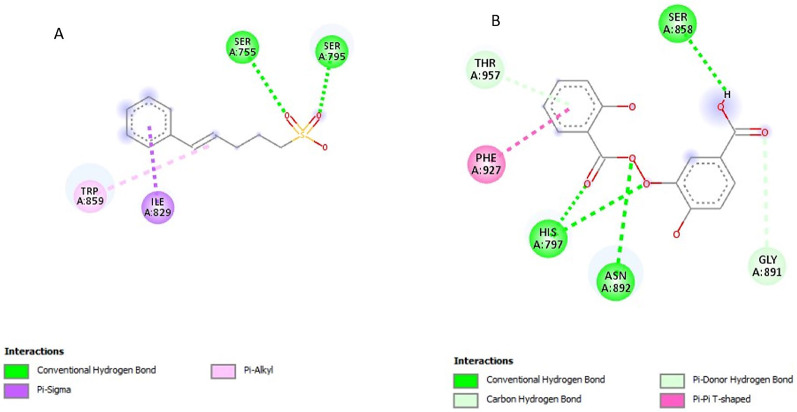
2D structures representing the binding interactions between **A**: **149253472** and **B**: **155204487** with *Pf*SUB2

**Fig. 10:**
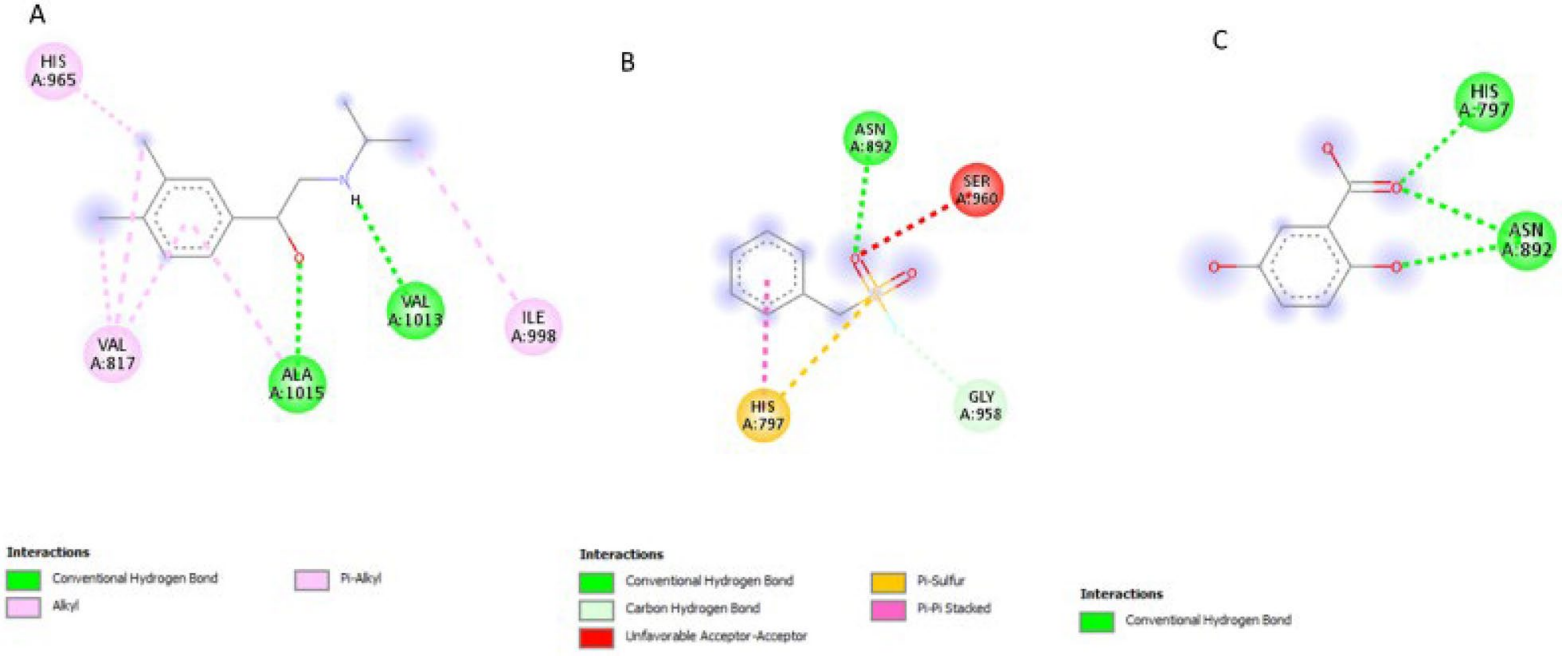
2D structures representing the binding interactions between **A**: **5806**, **B**: **4784**, and **C**: **19** with *Pf*SUB2

The interactions of the ligands in the active site of *Pf*SUB2 were visualised with the Discovery Studio 2021 client software. The interactions are shown in Table 4. The compound 154414021, a structure similar to PMSF, possesses 0 HBD and 4 HBA and exhibits the lowest binding affinity at −5.9 kcal/mol and is, therefore, the first hit. It possesses. It formed conventional hydrogen bonds with SER755 and SER795, Pi-Alkyl interactions with TRP859 and LYS776, and Pi-Sigma interactions with ILE829 (Fig. 7A). Compound 5919167 with 4 HBD and 5 HBA formed conventional hydrogen bonds with THR957 and GLY860, and Pi-Pi T-shaped with TRP859 (Fig. 7B). Compound 154074555 exhibited a binding affinity of −5.8 kcal/mol; it possesses 2 HBD and 7 HBA and formed conventional hydrogen bonds with GLY860, SER858, ASN892 and HIS797 and Pi-Pi T-shaped interaction with TRP859 (Fig. 8A). Compound 88759054 exhibited a binding affinity of −5.8 kcal/mol; it formed conventional hydrogen bonds with SER795, SER755 and GLN781, Pi-Alkyl interaction with LYS776 and Pi-Sigma interaction with ILE829 (Fig. 8B). Compound 149253472 formed conventional hydrogen bonds with SER755 and SER795, Pi-Alkyl interactions with TRP859, and Pi-Sigma interactions with ILE829 (Fig. 9A). Compound 155204487 with 3 HBD and 7 HBA exhibited a binding affinity of −5.7 kcal/mol and formed conventional hydrogen bonds with SER858, HIS797 and ASN892, carbon-hydrogen bonds with THR957 and GLY891 and Pi-Sigma interaction with PHE927 (Fig. 9B). Compound 5806 with 2 HBD and 2 HBA exhibited a binding affinity of −4.5 kcal/mol, formed conventional hydrogen bonds with VAL1013 and ALA1015, and Alkyl/Pi-Alkyl interactions with HIS965, VAL817 and ILE998 (Fig. 10A). Compound 4784 showed a binding affinity of −4.2 kcal/mol and possessing 0 HBD and 4 HBA formed conventional hydrogen bonds with ASN892, carbon-hydrogen bonds with GLY958, and Pi-Cation interaction with HIS797 (Fig. 10B). Compound 19 with 3 HBD and 4 HBA formed only conventional hydrogen bonds with HIS797 and ASN892 (Fig. 10C).

**Table 4:** Ligand interactions of selected compounds with *Pf*SUB2

S/*N*	Compound Codes	Hydrogen Bond Donors	Hydrogen Bond Acceptors	Interactions and Bond Lengths
1	154414021	0	3	Conventional Hydrogen Bond: SER755 and SER795 Pi-Alkyl: TRP859 and LYS776 Pi-Sigma: ILE829
2	5919167	4	5	Conventional Hydrogen Bond: THR957 and GLY860 Pi-Pi T-shaped: TRP859
3	154074555	2	7	Conventional Hydrogen Bond: GLY860, SER858, ASN892 and HIS797 Pi-Pi T-shaped: TRP859
4	88759054	1	3	Conventional Hydrogen Bond: SER795, SER755 and GLN781 Pi-Alkyl: LYS776 Pi-Sigma: ILE829
5	149253472	3	5	Conventional Hydrogen Bond: SER755 and SER795 Pi-Alkyl: TRP859 Pi-Sigma: ILE829
6	155204487	3	7	Conventional Hydrogen Bond: SER858, HIS797 and ASN892 Carbon-Hydrogen Bond: THR957 and GLY891 Pi-Sigma: PHE927
7	DCI	2	2	Conventional Hydrogen Bond: VAL1013 and ALA1015 Alkyl/Pi-Alkyl: HIS965, VAL817 and ILE998
8	PMSF	0	3	Conventional Hydrogen Bond: ASN892 Carbon-Hydrogen Bond: GLY958 Pi-Cation interaction: HIS797
9	DBH	3	4	Conventional Hydrogen Bonds: HIS797 and ASN892

The ligand interactions revealed that most of the compounds binding to the histidine active site of the protein (797–807) were similar to those of its inhibitor, PMSF. Only DCI and 5919167 bonded to the serine active site (958–1019).

### Molecular dynamics simulations of *Pf*SUB2

**Fig. 11:**
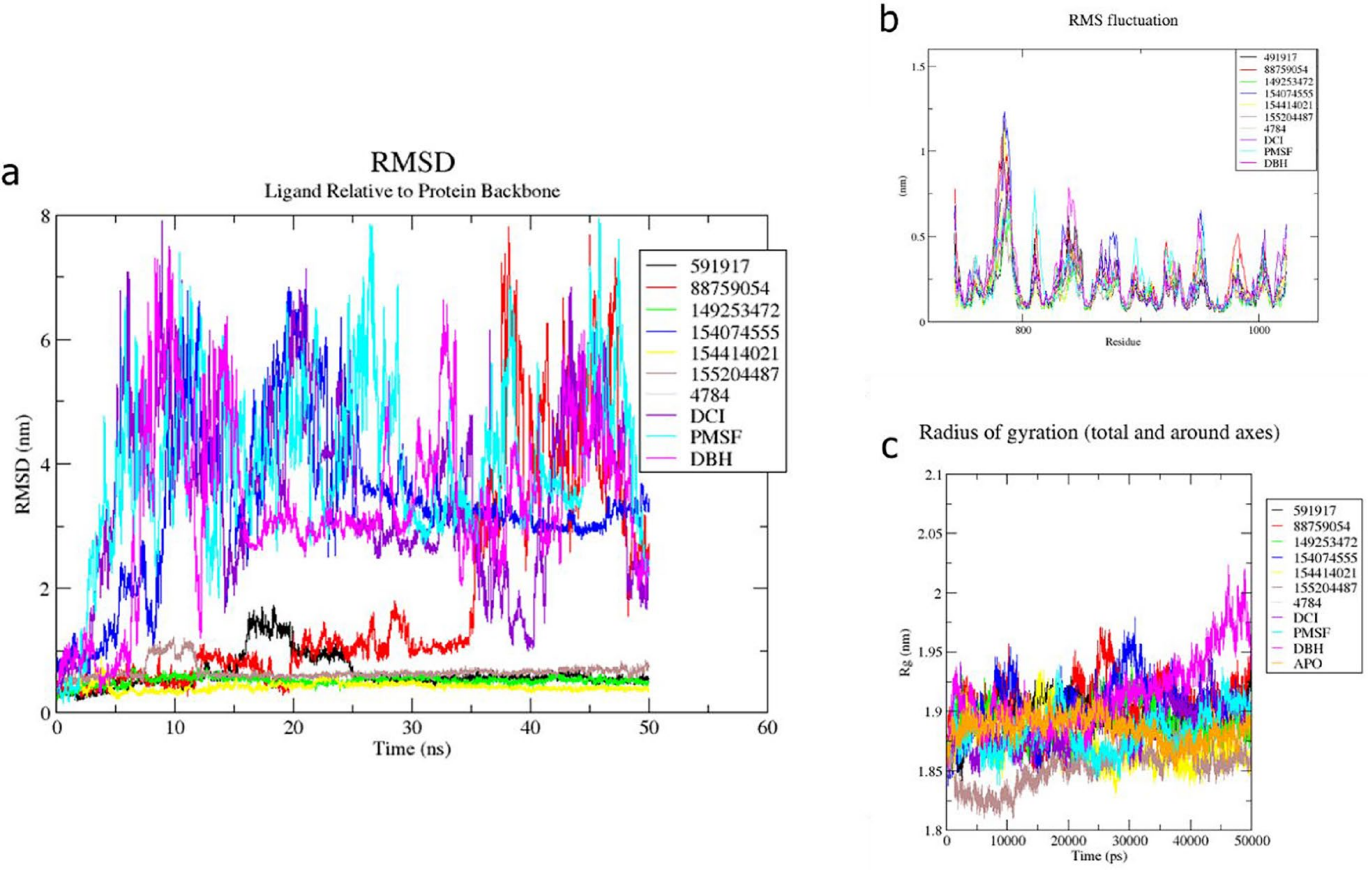
Molecular dynamics simulation analyses of protein-ligand complexes. (**a**) Root Mean Square Deviation (RMSD) of ligands relative to the protein backbone over a 50 ns simulation period, showing structural stability and binding persistence of each ligand. (**b**) Root Mean Square Fluctuation (RMSF) of protein residues in the presence of various ligands, highlighting local flexibility and potential ligand-induced stabilisation or destabilisation across the binding site region. (**c**) Radius of gyration (Rg) of the protein-ligand complexes over time, representing the compactness and structural integrity of the protein in each system during the simulation. Each system is colour-coded as indicated in the legend, with comparisons including control (APO), known inhibitors (e.g., PMSF, DBH), and test compounds.

The RMSD trajectories of the ligands relative to the protein backbone provided insights into the binding stability and conformational adaptability of the ligands within the active site during the 50 ns simulation. Four ligands, 5919167, 149253472, 154414021, and 155204487, demonstrated highly stable binding profiles, maintaining RMSD values consistently around 0.25 nm (Fig. 11a), indicating strong and persistent interactions with the binding pocket. Compound 5919167, although generally stable, exhibited a transient spike in RMSD between 15 ns and 25 ns, rising slightly above 1 nm, before stabilising again. This fluctuation may reflect a pose readjustment or induced-fit conformational change that allowed the ligand to optimize its interactions within the binding site. Ligand 88759054 initially remained stably bound, with an RMSD around 0.25–1.0 nm for the first 35 ns. However, it exhibited a series of spikes: a minor increase at 12 ns, a drop at 18 ns, followed by a larger spike between 20 and 30 ns. After 35 ns, its RMSD escalated significantly (beyond 6 nm), indicating that the ligand had dissociated from the binding pocket, possibly after sampling multiple unstable binding modes. In contrast, ligands 154074555, 4784, DCI, PMSF, and DBH displayed highly unstable trajectories throughout the simulation. These compounds showed erratic RMSD fluctuations, with several exceeding 3–6 nm, suggesting weak binding, lack of conformational restraint, or early egress from the binding site. Together, the RMSD data suggest that 5919167, 149253472, 154414021, and 155204487 are promising leads with favourable dynamic binding behaviour, while others either underwent significant rearrangement or failed to maintain stable interactions during the simulation period.

The RMS fluctuation analysis probes into the side-chain mobility in distinct regions of the protein, as revealed by the RMSF profiles (Fig. 11b). In general, rigid secondary‐structure elements remained stable (RMSF ≈ 0.2–0.3 nm), whereas surface loops (residues ∼760–1,030) exhibited variable fluctuations depending on the ligand. For instance, PMSF induced the largest increases in flexibility, peaking at ~ 1.5 nm around residue 800 and ~ 1.3 nm at residues 900 and 920, suggesting that this inhibitor perturbs multiple loop segments near the active site. Similarly, DBH caused pronounced spikes at ~ 1.2 nm around residue 850, ~ 1.1 nm near residue 950, and ~ 1.0 nm close to residue 1,010, indicating loop destabilization that may transiently disrupt protein–ligand contacts. Compound 88759054 displayed a single, prominent fluctuation of ~ 1.3 nm around residue 990 while remaining relatively stable elsewhere, implying a localized conformational adjustment in that loop. In sharp contrast, the four most stable ligands (5919167, 149253472, 154414021, 155204487) maintained uniformly low RMSF values (< 0.5 nm) across all residues, with only minor peaks (0.6–0.8 nm) at standard loop positions, consistent with their high binding persistence in the RMSD analysis. Intermediate behavior was observed for 154074555, 4784, and DCI, which induced moderate loop flexibility (RMSF ≈ 0.7–1.0 nm) without the extensive destabilization seen for PMSF or DBH. These differential RMSF patterns indicate that each ligand uniquely modulates loop mobility. PMSF and DBH produce pronounced loop disturbances, 88759054 perturbs a specific loop at residue 990, and top‐ranked small molecules preserve protein rigidity, thereby providing insight into how distinct chemotypes influence local protein dynamics.

Folding is crucial for protein structure and function. The folding of a protein can be determined by its level of compactness. In this work, the protein compactness for the various complexes was analyzed using Radius of Gyration plots (Fig. 11c), which describe the compactness and flexibility of the protein relative to its center. Overall, the 155204487, 154414021, and PMSF showed lower Rg scores compared to the APO (unbound protein), indicating that these compounds induce protein compactness in their bound state. Compactness may suggest stabilization of the protein in its bound state, which could trigger an inactive conformer. 5919167, 4784, and 149253472 demonstrated a plot comparable to the APO protein, indicating that 5919167, 4784, and 149253472 stabilize the protein in its inactive form. 88759054, 154074555, DCI, and especially DBH exhibited higher Rg scores (up to 2.5 nm), revealing that these complexes relaxed the protein to achieve a rather open structure.

### ADMET and drug-like properties of the selected compounds

The water solubility of the selected compounds ranged from − 5.987 to −1.821, while those of the control ranged from − 2.457 to −1.477. These values show the poor solubility of these selected compounds, which might lead to poor bioavailability of such compounds if administered as drugs. Solubility can, however, be enhanced by different methods such as particle size reduction, pH adjustment, hydrotropy, solid dispersion, salt formation, etc [37, 38]. The lipophilicity of the selected compounds ranged from − 0.0706 to 5.2422, while that of the control was 0.796 to 3.0248. An optimal lipophilicity value indicates a compound that balances solubility and permeability, ranging from 1 to 3. Most of the selected compounds have values within that range. Also, most of the compounds are neither P-glycoprotein substrates nor inhibitors (Table 5). Similarly, most of the compounds are neither substrates nor inhibitors of certain CYP variants (Table 6). The log BB values ranged from − 0.059 to 0.558 for the compounds and − 0.105 to 0.255. Considering that compounds with BBB crossing potential have values greater than 0, most of the compounds are deemed poorly distributed to the brain [39]. The excretion and toxicity properties (Table 7) indicated that none of the compounds are considered toxic according to AMES. At the same time, all showed relatively low values for the max tolerated doses in a range of 0.243–0.969 (log mg/kg/day). None of the compounds were considered inhibitors of hERG I and hERG II, thus indicating a reduced risk of fatal arrhythmia [40]. In summary, the selected compounds exhibit poor water solubility but mostly optimal lipophilicity, limited blood-brain barrier permeability, low predicted toxicity, and favorable excretion profiles, supporting their potential as safe drug candidates despite solubility challenges.

**Table 5:** The absorption properties of the selected hits

Compounds	Water Solubility (log M/L)	Lipophilicity	Intestinal Absorption	*P*-glycoprotein substrate	*P*-glycoprotein I inhibitor	*P*-glycoprotein II inhibitor	BBB Permeability (log BB)	CNS Permeability (log PS)
139974934	−4.979	3.9716	85.842	No	No	No	0.558	−2.532
139975082	−5.987	5.2422	81.952	No	Yes	No	0.552	−2.912
135307358	−2.886	2.1612	24.438	Yes	No	No	−1.027	−3.131
155204487	−2.848	1.9468	71.99	Yes	No	No	−1.137	−3.341
136630885	−1.821	1.3632	46.107	Yes	No	No	−1.162	−3.248
154414021	−2.993	2.4153	96.897	No	No	No	−0.059	−2.458
5919167	−2.859	2.672	51.204	Yes	No	No	−0.986	−2.421
154074555	−2.822	−0.0706	19.463	No	No	No	−0.499	−3.43
88759054	−2.517	1.9777	93.859	No	No	No	0.416	−2.428
149253472	−2.666	2.3678	93.404	No	No	No	0.349	−2.442
DCI	−2.457	3.0248	87.997	No	No	No	0.255	−1.708
DBH	−2.219	0.796	70.775	No	No	No	−0.757	−3.305
PMSF	−1.477	1.4859	93.866	No	No	No	−0.105	−1.809

**Table 6:** The metabolism properties of the hits

Compounds	CYP2D6 Substrate	CYP3A4 Substrate	CYP1A2 Inhibitor	CYP2C19 Inhibitor	CYP2C9 Inhibitor	CYP2D6 Inhibitor	CYP3A4 Inhibitor
139974934	No	Yes	No	Yes	No	No	No
139975082	No	Yes	No	Yes	No	No	No
135307358	No	No	No	No	No	No	No
155204487	No	No	No	No	No	No	No
136630885	No	No	No	No	No	No	No
154414021	No	Yes	No	No	No	No	No
5919167	No	No	No	No	No	No	No
154074555	No	No	No	No	No	No	No
88759054	No	No	No	No	No	No	No
149253472	No	No	No	No	No	No	No
DCI	No	No	Yes	No	No	Yes	No
DBH	No	No	No	No	No	No	No
PMSF	No	No	Yes	No	No	No	No

**Table 7:** Excretion and toxicity properties

Compounds	Total Clearance (log ml/min/kg)	AMES Toxicity	Max Tolerated Dose (log mg/kg/day)	hERG I Inhibitor	hERG II Inhibitor	Oral Rat Acute Toxicity (mol/kg)	Hepatotoxicity
139974934	0.283	No	0.482	No	No	3.888	Yes
139975082	0.437	No	0.243	No	No	4.145	Yes
135307358	0.369	No	0.45	No	No	2.223	Yes
155204487	0.397	No	0.666	No	No	3.02	No
136630885	0.21	No	0.433	No	No	2.279	No
154414021	0.926	No	0.406	No	No	2.172	No
5919167	0.191	No	0.595	No	No	3.089	Yes
154074555	0.783	No	0.915	No	No	1.99	No
88759054	0.061	No	0.66	No	No	2.114	No
149253472	0.075	No	0.561	No	No	2.199	No
DCI	0.925	No	0.728	No	No	3.007	No
DBH	0.442	No	0.787	No	No	2.267	No
PMSF	0.26	No	0.969	No	No	2.283	No

## Conclusion

This study focused on identifying potential inhibitors of *Pf*SUB2 and *Pf*TRAMP as potential antimalarial therapy using in silico methods. Compared with the reference inhibitors, the binding energy of the compounds was better. Although the compounds didn’t adhere well to drug-likeness rules, they can be optimised during hit-to-lead optimisation processes. This study highlights 139974934 and 154414021 as good inhibitors and potential antimalarials targeting *Pf*TRAMP and *Pf*SUB2. It also highlights 155,204,487 as a compound with dual antimalarial target potential. In vitro and in vivo studies should be undertaken to validate these results.

## Electronic supplementary material

Supplementary Material 1

## Data Availability

All datasets generated and/or analyzed during the current study are included in this published article and its supplementary information files. Additional data are available from the corresponding author upon reasonable request.
